# Treatment outcome and clinical characteristics of HER2 mutated advanced non‐small cell lung cancer patients in China

**DOI:** 10.1111/1759-7714.13317

**Published:** 2020-01-23

**Authors:** Fei Xu, Guangjian Yang, Haiyan Xu, Lu Yang, Weini Qiu, Yan Wang

**Affiliations:** ^1^ Department of Medical Oncology, National Cancer Center/National Clinical Research Center for Cancer/Cancer Hospital Chinese Academy of Medical Sciences and Peking Union Medical College Beijing China; ^2^ Department of Comprehensive Oncology National Cancer Center/National Clinical Research Center for Cancer/Cancer Hospital and Chinese Academy of Medical Sciences and Peking Union Medical College Beijing China; ^3^ Haalthy Beijing China

**Keywords:** Chemotherapy, HER2/ERBB2 mutation, non‐small cell lung cancer, targeted therapy

## Abstract

**Background:**

HER2 mutation is found in 1%–2% of lung cancer patients. Studies comparing chemotherapy to HER2‐TKIs are limited. This study aimed to investigate the molecular and clinical patterns of HER2 mutations in advanced non‐small cell lung cancer (NSCLC), and compare the different outcomes between chemotherapy and HER2‐TKIs.

**Methods:**

Advanced or recurrent non‐small cell lung cancer patients with de novo HER2 mutations (*N* = 75) were included in this study. Molecular information, clinical features, and treatment outcomes were retrospectively collected from a web‐based patient registry and hospital chart review.

**Results:**

Between October 2012 and December 2018, 65 patients with in‐frame insertion mutations, eight with point mutations and two with gene amplification were found. The most common subtypes of insertion mutations were A775_G776insYVMA, G776delinsVC, and V777_G778insGSP. HER2 mutated patients were mostly young‐aged, females, never or light smokers, with adenocarcinoma. Chemotherapy achieved better outcomes than HER2‐TKIs (median PFS: 5.5 vs. 3.7 months in the first‐line setting and 4.2 vs. 2.0 months in the second‐line setting, *P* = 0.001 and 0.031, respectively). In particular for the most common subtype, YVMA insertions, PFS was significantly longer in chemotherapy than HER2‐TKIs both in the first‐line (6.0 vs. 2.6 months, *P* = 0.008) and the second‐line (4.2 vs. 2.6 months *P* < 0.001).

**Conclusions:**

HER2 mutated lung cancer patients were younger, mostly females, never or light smokers, with histologically diagnosed adenocarcinomas. Compared with afatinib, chemotherapy might bring more benefit to HER2 mutated advanced lung cancer patients, especially the most common type of HER2 exon 20 insertions, A775_G776insYVMA subtype.

**Key points:**

Chemotherapy achieved better outcomes than afatinib for Chinese HER2 mutated advanced NSCLC patients, especially for the most common subtype, YVMA insertions.

## Introduction

Lung cancer has the highest mortality among all malignancies. The five‐year survival rate of lung cancer patients is only 19%. In 2019, an estimated 228 150 people were predicted to be newly diagnosed with lung and bronchus tumor in the United States, with estimated deaths of 142 670.^1^

Therapeutic strategies for lung cancer has been rapidly progressing. Targeted therapy has provided a positive outcome for lung cancer patients with epidermal growth factor receptors (EGFR), anaplastic lymphoma kinase (ALK), ROS proto‐oncogene 1 receptor tyrosine kinase (ROS1) and B‐Raf proto‐oncogene serine/threonine kinase (BRAF) mutations.^2^, ^3^, ^4^, ^5^

In 2004, Erb‐B2 Receptor Tyrosine Kinase 2(HER2/ERBB2) kinase domain mutations were identified in non‐small cell lung cancer (NSCLC).^6^ It has been reported that HER2 mutations could promote cell proliferation and motility in vitro and was essential in tumorigenesis and maintenance in vivo.^7^, ^8^ HER2 mutations in NSCLC were dominated by in‐frame insertions in exon 20 of HER2 kinase domain, with the most common subtype being A775_G776insYVMA.^9^ For advanced NSCLC patients harboring HER2 mutations, the median overall survival was reported to be approximately 19 to 22.9 months.^10^, ^11^

HER2 mutations and potential therapeutic options make the study of survival pathways appealing targets. Great benefit has been seen in breast cancer or gastric cancer patients treated with Trastuzumab, a monoclonal antibody against HER2.^12^ Several HER2‐tyrosine kinase inhibitors (TKIs) have also been found to be effective in NSCLC cell lines with HER2 mutation.^8^, ^13^, ^14^ However, response to HER2‐TKIs has been reported to be unsatisfactory in HER2 mutated NSCLC patients.^9^, ^15^, ^16^, ^17^

Until now, there has been no clear evidence or guidelines for treatment of HER2 mutated NSCLC patients. In the real clinical scenario, treatment varies from chemotherapy to targeted therapy, even immunotherapy. Here, we retrospectively summarized information on HER2 mutated advanced NSCLC patients from China and analyzed molecular patterns, clinical characteristics and treatment outcome.

## Methods

### Study design

A retrospective, nationwide study was designed to clarify the clinical features of HER2 mutated advanced non‐small cell lung cancer patients, as well as the difference between two main treatment regimens, chemotherapy and HER2 targeted therapy.

### Patients’ characteristics

Between October 2012 and December 2018, information on 75 advanced NSCLC patients with HER2 mutations were collected from a web‐based patient registry and hospital chart review, including patients from the National Cancer Center/National Clinical Research Center for Cancer/Cancer Hospital, Chinese Academy of Medical Sciences and Peking Union Medical College (information of patients' frequently visited hospitals is presented in Table S1). Clinicopathological characteristics collected for analysis included gender, age at diagnosis, smoking history, histologic subtypes, clinical TNM stage, and variables of insertion mutations. Therapeutic outcomes were documented and retrospectively collected. Disease recurrence and survival outcomes were recorded according to follow‐up clinic visits or telephone calls. This study was approved by the Ethics Committee of the National Cancer Center/National Clinical Research Center for Cancer/Cancer Hospital, Chinese Academy of Medical Sciences and Peking Union Medical College in accordance with the declaration of Helsinki protocol. Written informed consent was obtained from all patients.

## HER2 mutant identifications

HER2 mutant identifications (exon17‐26) were performed by next‐generation sequencing. Peripheral blood or tumor samples were used.

## Statistical analysis

The Kaplan‐Meier method was used to calculate the curves for PFS. The Cox proportional hazards regression model was used to evaluate the impact of collected clinical variables on PFS. Significant differences were determined by the log‐rank test. *P*‐values less than 0.05 was considered statistically significant. Statistical analyses were performed using SPSS software, version 22.0 software (IBM Corp., Armonk, NY, USA).

## Results

**Figure 1:**
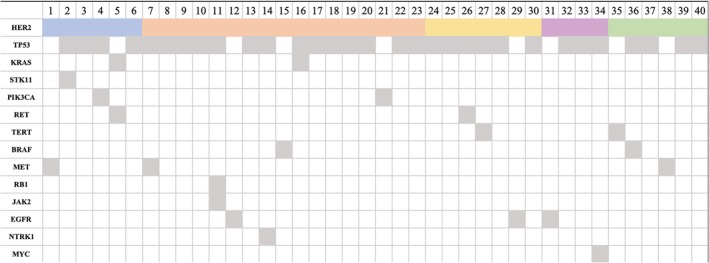
Co‐mutation patterns of HER2 mutated lung cancer. A total of 40 patients with advanced lung cancer who harbored more than one mutation were included. The most frequently mutated gene was TP53 mutation (77.5% patients). Note: blue box: HER2 point mutation; orange box: A775_G776insYVMA; yellow box: G776delinsVC; purple box: V777_G778insGSP; green box: other HER2 mutations.

A total of 75 cases were diagnosed with de novo HER2 mutated advanced lung cancer, including 65 with in‐frame insertion mutations, eight with point mutations and two with gene amplifications. Among them, one squamous cell lung carcinoma with HER2 amplification was found. There were mainly three common subtypes of insertions detected in HER2 exon 20: c.2326_2327insTGT, p.Gly776delinsValCys, simplified as G776VC; c.2313_2324dupATACGTGATGGC, p.Ala775_Gly776insTyrValMetAla, simplified as YVMA; c.2330_2331insGGGCTCCCC, p.Val777_Gly778insGlySerPro, simplified as GSP. Patient numbers in each subtype were 13, 38 and seven, respectively. HER2 mutation was mutually exclusive with other driver mutations of lung cancer in most cases, except for two patients harboring EGFR exon20 co‐mutations. As shown in Fig. 1, 40 HER2 mutated lung cancer patients also harbored other mutations in next‐generation sequencing (NGS) results, of which 31 patients had TP53 co‐mutations.

Among 75 patients, females represented the majority with only 23 male patients. Median age was 57 (range: 31–78). A total of 21 patients had a smoking history, but only five were heavy smokers. In 30 patients with documentary histological subtype information, half were poorly differentiated, the other half were moderately or well differentiated. All patients had advanced or recurrent cancers, and nine were unresectable stage IIIB (locally advanced) patients. Metastasis was also investigated. For 66 patients with stage IV, 34 had oligometastasis, and metastasis to brain, liver, bone, and lung was 12, 5, 22, and 25, respectively. All pretreatment clinical characteristics are listed in Table 1. For univariate analysis of progression‐free survival of first‐line treatment (PFS1), all clinical characters mentioned above were analyzed. None had a significant effect on the duration of response to drugs used in the first‐line setting. Table 2 shows the detailed results.

**Table 1:** Clinical characteristics of HER2 mutated advanced lung cancer patients

Parameters	Groups	*N* (%)
Age	<65	57 (76.0)
	≥65	18 (24.0)
Sex	Male	23 (30.7)
	Female	52 (69.3)
Smoking history	Never	54 (72.0)
	Light smoker	16 (21.3)
	Heavy smoker	5 (6.7)
Stage	IIIB	9 (12.0)
	IV	66 (88.0)
Histology	Poorly differentiated adenocarcinoma	15 of 30 (50.0)
	Moderately or well differentiated adenocarcinoma	15 of 30 (50.0)
	Squamous cell carcinoma	1 of 75 (1.3)
Metastasis number	Oligometastasis	34 of 66 (51.5)
	Multiorgan metastasis	32 of 66 (48.5)
Metastasis sites	Brain	12 of 66 (18.1)
	Lung	25 of 66 (37.9)
	Liver	5 of 66 (7.6)
	Bone	22 of 66 (33.3)

If not specified, the percentage was calculated by the number of patients in the subgroup divided by the whole.

**Table 2:** Univariate analysis of clinical features on treatment responses (Cox regression model) which showed no significant impact of all clinical parameters on first‐line treatment outcome

Clinical parameters	B	HR (CI)	*P*‐value
Age	0.576	1.778 (0.973–3.249)	0.061
Sex	0.367	1.443 (0.803–2.593)	0.220
Smoking history	−0.295	0.744 (0.491–1.127)	0.163
Metastasis number	0.038	1.038 (0.619–1.740)	0.886
Brain metastasis	0.459	1.582 (0.815–3.069)	0.175
Stage	−0.352	0.703 (0.217–2.273)	0.556
Histology	0.208	1.231 (0.550–2.755)	0.613
TP53 co‐mutation	−0.173	0.841 (0.497–‐1.423)	0.841

**Figure 2:**
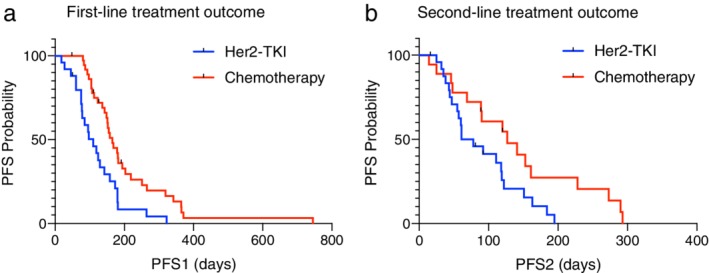
Treatment response among HER2 mutated lung cancer patients as a whole. Treatment response was different between HER2‐targeted TKIs and chemotherapy, both in (**a**) first‐line and (**b**) second‐line settings.

**Figure 3:**
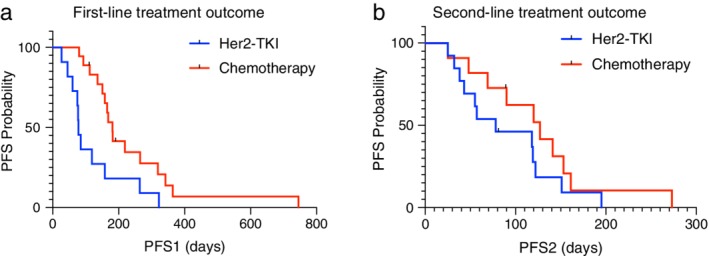
Treatment response difference in YVMA subtype of HER2 exon 20 insertion mutated lung cancer patients. Treatment response was different between HER2‐targeted TKIs and chemotherapy in first‐line (a) and second‐line (b) settings in YVMA subtype of HER2 exon 20 insertion mutated lung cancer patients.

**Figure 4:**
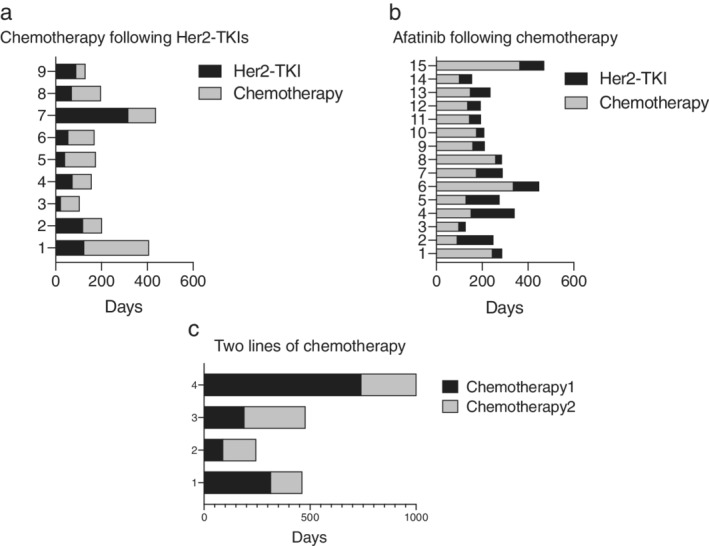
Progression‐free survival of different first‐ and second‐line treatment sequential. When taken together, whether applying HER2 targeted TKIs or chemotherapy as the first‐line treatment, the overall progression‐free survival (PFS1 + PFS2) was similar, while patients using two lines of chemotherapy received more benefit. Nevertheless, only four patients chose the two‐line chemotherapy regimen.

On treatment analysis, apart from eight patients without available treatment information and one squamous cell lung cancer patient, 66 patients with treatment outcomes were analyzed. The median line of treatment for advanced patients was three. Afatinib, pyrotinib and poziotinib were regarded as one group of treatment named HER2‐TKI therapy. Chemotherapy included pemetrexed and platinum with or without anti‐VEGF agents. Others included mono antivascular agents such as anlotinib and immune checkpoint inhibitors. Only first‐ and second‐line treatment outcome were included in the analysis. In the first‐line setting, 37 patients received chemotherapy and 29 patients were treated with targeted therapy (22 with afatinib, four with first generation EGFR‐TKIs, one with pyrotinib, one with poziotinib, and one with trastuzumab). Patients treated with chemotherapy had longer PFS1 than those who received HER2‐TKIs. The median PFS1 of HER2‐TKIs and chemotherapy was 3.7 months (95% CI 2.3 to 5.0 months) and 5.5 months (95% CI 4.5 to 6.5 months), *P* = 0.001. Similar difference was seen in the second‐line treatment as shown in Fig. 2a,b. The median progression‐free survival of second‐line treatment (PFS2) of chemotherapy and HER2‐TKIs was 4.2 months (95% CI 2.2 to 6.3 months) and 2.0 months (95% CI 0.8 to 3.3 months), *P* = 0.031. In subgroup analysis, YVMA, the most common subtype of HER2 exon 20 insertions, possessed similar treatment response patterns compared to the population as a whole. As shown in Fig. 3a,b, the median PFS1 for chemotherapy and HER‐TKIs was 6.0 months (95% CI 5.3 to 6.8 months) and 2.6 months (95% CI 2.2 to 3.0 months) in YVMA subgroup, *P* = 0.008. The median PFS2 for chemotherapy and HER‐TKIs was 4.2 months (95% CI 2.4 to 6.1 months) and 2.6 months (95% CI 0.1 to 5.1 months) in this subgroup, *P* < 0.001. While for non‐YVMA insertions, chemotherapy provided 0.8 months longer PFS than HER2‐TKIs, but there was no significant difference seen between the two groups (*P* = 0.084). When taken together, survival (PFS1 + PFS2) of HER2‐TKIs plus chemotherapy were not affected by different order of the two agents (*P* = 0.263), but was shorter than two lines of chemotherapy as illustrated in Fig. 4.

## Discussion

HER2 mutations in our cohort included point mutations, in‐frame insertions, which accounted for the majority of cases, and gene amplification was seen in one patient with squamous cell lung cancer. Mutation in squamous cell lung cancer is rather rare, and HER2 mutation has not previously been reported. Among HER2 exon 20 insertions, there were A775_G776insYVMA, G776delinsVC, V777_G778insGSP etc, with the most common type being YVMA, in accordance with previous findings. We also paid attention to the co‐mutations of HER2, and for patients harboring exon 20 insertions, TP53 was the most common co‐mutation. However, limited by various NGS platforms from different gene companies, we could not summarize the relevance between mutation abundance and clinical characteristics of those patients.

Consistent with former studies, HER2 mutated patients in our cohort were mainly females, never or light smokers, with poorly or moderately differentiated adenocarcinoma.^10^ They were younger, with more than three quarters of patients aged less than 65 years old.

In terms of treatment outcome, only first‐ and second‐line treatment were analyzed. In the first‐line settings, our data showed that HER2 targeted therapy had an inferior outcome compared with the standard of care chemotherapy. This was contrary to previous studies. One study including 24 HER2 exon 20 insertion lung cancer patients revealed that the overall survival of targeted therapy was longer than nontargeted agents, with 2.1 years and 1.4 years, respectively.^18^ Eng *et al*. reported 38 cases of HER2 mutated patients in which the PFS of HER2‐TKIs was 2.2 months, with 5.2 months for first‐line treatment and 1.8 months in later lines. The overall median PFS of chemotherapy was 4.3 months, with pemetrexed plus platinum or bevacizumab possessing the longest period of 6.2 months.^19^ In our study, the PFS of first‐line HER2‐TKIs and chemotherapy was longer than previously reported. This was possibly because pemetrexed plus platinum was used in the majority of patients as their chemotherapy regimen.

With regard to the analysis of subtype, within the YVMA subtype, HER2‐TKIs had a shorter PFS than chemotherapy. This finding was again contrary to the findings of previous studies. Peters *et al*. stated that the time to treatment failure (TTF) was 9.6 months in YVMA subtype, much longer than the 2.9 months of all patients.^20^ Another study compared the different responses to first‐line chemotherapy, reporting that YVMA had a PFS of 0.9 month shorter than the overall survival.^21^ Our results indicated that for YVMA patients, chemotherapy was a better choice in first‐line treatment. Since patients with YVMA in our cohort had been exposed to both treatments, our results might represent a more direct conclusion. In subtypes other than YVMA, there was no significant difference seen between chemotherapy and HER2‐TKIs.

The HER2‐TKIs used as first‐ or second‐line treatment in our patient cohort were mainly afatinib. This is partly because of the availability of legal drugs in China. HER2‐TKIs such as poziotinib, dacotinib and pyrotinib are taken orally and rarely used, and this also applies to trastuzumab and T‐DM 1 which are used as intravenous agents in health care centers. However, this does not mean that these drugs are invalid for HER2 mutated lung cancer patients. For HER2 amplification positive lung cancer patients, adding trastuzumab to their chemotherapy regimen did not seem to bring more clinical benefit.^22^ However, it has been reported that trastuzumab or T‐DM 1 had an objective response rate (ORR) of 50.9% and PFS of 4.8 months in HER2 exon 20 mutated patients.^17^ In another cohort involving seven HER2 exon 20 insertion patients, five patients achieved a partial response or stable disease on T‐DM1 treatment.^23^ This could mean that trastuzumab or T‐DM1 might be beneficial to a minority of patients. Among small molecule TKIs, afatinib was most widely used in China in recent years, with PFS ranging from 2.9 to 6 months.^20^, ^24^, ^25^, ^26^, ^27^ Poziotinib, neratinib and pyrotinib had similar PFS, with 4.5–5.5 months, 5.5 and 6.4 months, respectively.^9^, ^28^, ^29^ Dacomitinib and Osimertinib had weaker effects on this population, either with a short PFS or being invalid in cell line experiments.^15^, ^30^

Our study collected information from widespread geographical parts of China, discussing the different outcomes of treatment among HER2 mutated advanced lung cancer patients. We concluded that for HER2 mutated advanced non‐small cell lung cancer patients, chemotherapy would bring more benefit than afatinib, especially in the most common subtype of exon 20 insertions. Nevertheless, limited by the retrospective information collection, some important parameters were missing. In patients lacking tumor tissue samples, we accepted NGS outcomes from peripheral blood samples. Treatment varied significantly and it was no easy task to categorize them appropriately. Patients' samples were sent to different centers for analysis which meant that we could not summarize the relevance between mutation abundance and response to treatment. A prospective study is warranted to explore the efficacy of chemotherapy and different TKIs in HER2 mutated lung cancer patients, and a standardized platform to test the mutant alleles would be more convincing.

In conclusion, our study revealed that the most common HER2 mutations in advanced lung cancer were exon 20 insertions. HER2 mutated lung cancer patients were younger, mostly females, never or light smokers, and histologically adenocarcinoma dominated. Compared to afatinib, chemotherapy might bring more benefit to patients with HER2 mutated advanced lung cancer, especially the most common type of HER2 exon 20 insertions, A775_G776insYVMA.

## Disclosure

No authors report any conflict of interest.

## Supporting information


**Table S1** Information of patients' frequently visited health care centers
