# Supplementary material for: Treatment outcome and clinical characteristics of HER2 mutated advanced non‐small cell lung cancer patients in China
Source: Thorac Cancer. 2020 Jan 23;11(3):679–85. doi: 10.1111/1759-7714.13317 (PMC7049517; doi:10.1111/1759-7714.13317)
Supplement: Supplementary file 1 — Table S1 Information of patients' frequently visited health care centers [file TCA-11-679-s001.docx]

**Supplement table 1 Information of patients’ frequently visited health care centers**

| No. | Frequently Visited Hospitals |
| --- | --- |
| 1 | CAMS ^†^ |
| 2 | CAMS |
| 3 | West China Hospital of Sichuan University |
| 4 | CAMS |
| 5 | CAMS |
| 6 | Guangdong Provincial People's Hospital |
| 7 ^‡^ | Qiqihaer, Heilongjiang province |
| 8 | The First Hospital Affiliated to AMU (Southwest Hospital) |
| 9 | The General Hospital of Shenyang Military Region |
| 10 | CAMS |
| 11 | Ningbo Hospital of Zhejiang University |
| 12 | Hunan Cancer Hospital |
| 13 | Tianjin Medical University Cancer Center & Hospital |
| 14 | Jiangxi Provincial Cancer Hospital |
| 15 | Sun Yat-Sen University Cancer Center |
| 16 | Jiangsu Province Hospital |
| 17 | Shanghai Chest Hospital |
| 18 | Shanghai Chest Hospital |
| 19 | Sun Yat-Sen University Cancer Center |
| 20 | Tianjin Medical University Cancer Center & Hospital |
| 21 | Ruijin Hospital, Shanghai Jiao Tong University School of Medicine |
| 22 | The First Bethune Hospital of Jilin University |
| 23 | Zhongshan Hospital, Fudan University, |
| 24 | CAMS |
| 25 | Shanghai Chest Hospital |
| 26 | The First Affiliated Hospital, Zhejiang University |
| 27 | Fujian Cancer Hospital, Fujian Medical University Cancer Hospital |
| 28 | Sichuan Provincial People’s Hospital |
| 29^‡^ | Shenyang, Liaoning Province |
| 30 | CAMS |
| 31 | Hunan Cancer Hospital |
| 32 | Affiliated Hospital of Shandong Medical College |
| 33 | Xiangya Hospital, Central South University |
| 34 | The Affiliated Tumor Hospital of Harbin Medical University |
| 35 | CAMS |
| 36 | CAMS |
| 37 | CAMS |
| 38 | CAMS |
| 39 | Tongji Hospital, Huazhong University of Science and Technology |
| 40 | CAMS |
| 41 | Qilu Hospital of Shandong University |
| 42 | CAMS |
| 43 | First Affiliated Hospital of Zhengzhou University |
| 44 | Linyi Central Hospital |
| 45 | Shandong Cancer Hospital |
| 46 | Cancer Center of Guangzhou Medical University |
| 47 | Shanghai Chest Hospital |
| 48 | CAMS |
| 49 | CAMS |
| 50 | Affiliated Tumor Hospital of Guangxi Medical University |
| 51 | The First Affiliated Hospital, Zhejiang University |
| 52 | First Affiliated Hospital of Zhengzhou University |
| 53 | CAMS |
| 54 | Shandong Cancer Hospital |
| 55 | CAMS |
| 56 | The Second Xiangya Hospital of Central South University |
| 57 | Tangshan People’s Hospital |
| 58 | The First People Hospital of Jingzhou |
| 59 | CAMS |
| 60 | CAMS |
| 61 | Jilin Cancer Hospital |
| 62 | Yichang Hospital of Traditional Chinese Medicine |
| 63 | CAMS |
| 64 | The Second Xiangya Hospital of Central South University |
| 65 | CAMS |
| 66 | CAMS |
| 67 | The First Affiliated Hospital, Zhejiang University |
| 68 | Peking University First Hospital |
| 69 | People’s Hospital of Juxian |
| 70 | CAMS |
| 71 | Jiangsu Province Hospital of Chinese Medicine |
| 72 | Hunan Cancer Hospital |
| 73 | Renji Hospital, School of Medicine, Shanghai Jiao Tong University |
| 74 | First Affiliated Hospital of Zhengzhou University |
| 75 | CAMS |

†CAMS: National Cancer Center/National Clinical Research Center for Cancer/Cancer Hospital, Chinese Academy of Medical Science and Peking Union Medical College.

‡Information of frequently visited hospital was not provided. Only location of the patient’s home was presented here.
